# Evaluation of a simple method for testing aztreonam and ceftazidime-avibactam synergy in New Delhi metallo-beta-lactamase producing Enterobacterales

**DOI:** 10.1371/journal.pone.0303753

**Published:** 2024-05-17

**Authors:** Salman Khan, Arghya Das, Deepali Vashisth, Anwita Mishra, Ashima Jain Vidyarthi, Raghav Gupta, Nazneen Nahar Begam, Babita Kataria, Sushma Bhatnagar

**Affiliations:** 1 Department of Microbiology, National Cancer Institute, All India Institute of Medical Sciences (Jhajjar-campus), New Delhi, India; 2 Department of Microbiology, All India Institute of Medical Sciences, Madurai, India; 3 Department of Microbiology, Vallabhbhai Patel Chest Institute, New Delhi, India; 4 Department of Microbiology, Mahamana Pandit Madan Mohan Malviya Cancer Centre and Homi Bhabha Cancer Hospital, Varanasi, Uttar Pradesh, India; 5 Department of Oncoanesthesia and Palliative Medicine, National Cancer Institute, All India Institute of Medical Sciences (Jhajjar-Campus), New Delhi, India; 6 Department of Infectious Diseases, Institute of Post-Graduate Medical Education and Research, Kolkata, India; 7 Department of Medical Oncology, National Cancer Institute, All India Institute of Medical Sciences (Jhajjar-Campus), New Delhi, India; Tribhuvan University, NEPAL

## Abstract

NDM-producing carbapenem-resistant bacterial infections became a challenge for clinicians. Combination therapy of aztreonam and ceftazidime-avibactam is a prudent choice for these infections. However, there is still no recommendation of a practically feasible method for testing aztreonam and ceftazidime-avibactam synergy. We proposed a simple method for testing aztreonam and ceftazidime-avibactam synergy and compared it with reference broth micro-dilution and other methods. Carbapenem-resistant Enterobacterales clinical isolates were screened for the presence of the NDM gene by the Carba R test. NDM harbouring isolates were tested for aztreonam and ceftazidime-avibactam synergy by broth microdilution (reference method), E strip-disc diffusion, double disc diffusion, and disc replacement methods. In the newly proposed method, the MHA medium was supplemented with ceftazidime-avibactam (corresponding to an aztreonam concentration of 4μg/ml). The MHA medium was then inoculated with the standard inoculum (0.5 McFarland) of the test organism. An AZT disc (30 μg) was placed on the supplemented MHA medium, and the medium was incubated overnight at 37°C. Aztreonam zone diameter on the supplemented MHA medium (in the presence of ceftazidime-avibactam) was compared with that from a standard disc diffusion plate (without ceftazidime-avibactam), performed in parallel. Interpretation of synergy was based on the restoration of aztreonam zone diameter (in the presence of ceftazidime-avibactam) crossing the CLSI susceptibility breakpoint, i.e., ≥ 21 mm. Of 37 carbapenem-resistant NDM-producing isolates, 35 (94.6%) were resistant to aztreonam and tested synergy positive by the proposed method. Its sensitivity and specificity were 97.14% and 100%, respectively. Cohen’s kappa value showed substantial agreement of the reference method with the proposed method (κ = 0.78) but no other methods. The proposed method is simple, easily interpretable, and showed excellent sensitivity, specificity, and agreement with the reference method. Therefore, the new method is feasible and reliable for testing aztreonam synergy with avibactam in NDM-producing Enterobacterales.

## Introduction

In 2008, New Delhi metallo-beta-lactamase (NDM) was reported for the first time from a *Klebsiella pneumoniae* isolate causing urinary tract infection in a Swedish patient who visited New Delhi, India [1]. As the name suggests, it belongs to a metallo-beta-lactamase (MBL) or Ambler class B beta-lactamase enzymes, which can hydrolyze most of the beta-lactam group of antibiotics [2] except a few like cefiderocol, which is still not widely available across nations [3]. Owing to the unique structure of the MBL enzymes and the nature of zinc ligands and catalytic mechanisms, only a few inhibitors could be successfully designed to tackle these enzymes [4]. Furthermore, plasmid-mediated dissemination of the MBL, particularly blaNDM, plays an instrumental role in the rapid evolution of carbapenem-resistant Gram-negative bacteria, causing havoc in hospital settings [5]. The global burden of NDM-producing bacteria is primarily distributed in Asia (58.15%), with China and India as major contributors, followed by Europe (16.8%) and the American continents (10.8%), respectively [6]. The financial implications due to NDM are evident from a hospital report in the Netherlands with an estimated cost of $804,263 or €653,801 (12% of the total budget allocated that year for medical microbiology and infection prevention) attributed to a single outbreak of NDM-producing *K*. *pneumoniae* over three months in the year 2015 [7]. Undoubtedly, this enormous financial stress from treating and preventing NDM-producing superbugs is a grave concern for developing economies like India.

Fortunately, aztreonam (AZT), a monobactam, demonstrates good stability against all MBLs, and thus, it emerges as a prudent choice for treating infections caused by NDM-producing bacteria [6]. However, this drug must be protected from the AmpC and ESBL (extended-spectrum beta-lactamases) enzymes, often co-produced in NDM-producing Gram-negative Enterobacterales [8]. On the other hand, avibactam (AVI), a beta-lactamase inhibitor, has a broad spectrum of activity, neutralizing Ambler class A (KPC, ESBL), class C (AmpC) enzymes and some class D (OXA-48-like) enzymes [9]. Since AVI in a formulation with ceftazidime (CAZ) is widely available, combining CAZ-AVI with AZT becomes a potential treatment option against Gram-negative bacteria. In the year 2022, the Indian Council of Medical Research (ICMR), in its guidelines, has recommended using the above combination against NDM-producing Enterobacterales [10]. However, it is imperative to demonstrate AZT synergy in the presence of AVI in an in-vitro test before using the above regimen in patients [10]. There is no consensus or standard recommendation for a simple synergy testing method for the above-mentioned drug combination except broth microdilution (BMD) with checkerboard assay, which is labour-intensive for a busy routine diagnostic microbiology laboratory. A handful of synergy testing methods like the E-strip-disc diffusion method, E-strip stacking method, and E-strip cross method exist, which are not very labour-intensive but highly subjective and have variable accuracy [11].

In the present study, we proposed and evaluated a simple, cost-effective, and easily interpretable method to demonstrate AZT-AVI synergy against NDM-producing Enterobacterales.

## Materials and methods

### Ethics statement

The study protocol (Ref. No.: AIIMSA00270) was approved by the Institute Ethics Committee, All India Institute of Medical Sciences, New Delhi. The study involved experiments on already archived bacterial strains. The clinical isolates were fully anonymized and annotated by unique identification numbers. The investigators of this study had no access to data on patients from whom these clinical isolates were recovered during routine microbiological diagnostic testing.

Patients ’ consents were not obtained since the present study involved retrospective analysis of already archived and fully anonymized bacterial isolates.

### Study centre

The study was conducted in the Microbiology unit of the National Cancer Institute, Jhajjar campus under All India Institute of Medical Sciences, New Delhi, India.

### Bacterial strains and their selection for the experiments

The carbapenem-resistant Enterobacterales strains included in this study were initially isolated from different clinical specimens (pus, body fluids, blood, tissue, urine) of cancer patients admitted in other hospital wards from July 01, 2022, to December 15, 2022. Identification and susceptibility testing of the isolates was carried out with BD Phoenix^™^ M50 Automated Microbiology System using NMIC/ID-55 panels (Becton Dickinson, USA).

All the archived isolates in stock cultures were used in the experiments of this study during January and February 2023. Isolates were revived in fresh culture from stocks before the experimentations.

### Screening for carbapenemase determinants

Carbapenem-resistant Enterobacterales isolates were screened for common carbapenemases encoding genes with the Xpert Carba-R test in the Gene Xpert Dx System (Cepheid, Sunnyvale, CA, USA). Enterobacterales isolates, harbouring *bla*_*NDM*_, were selected and tested further for the study.

### Susceptibility testing by Kirby Bauer disc diffusion method

The selected *bla*_*NDM*_ harbouring Enterobacterales isolates were further tested by Kirby Bauer disc diffusion tests with CAZ (30μg), AZT (30μg), CAZ-AVI (30 μg /20 μg), imipenem (10μg), ertapenem (10μg) and meropenem (10μg) discs (HiMedia Laboratories, India). The results were interpreted as per zone diameter breakpoints described in the performance standards of antimicrobial susceptibility testing by the Clinical and Laboratory Standards Institute (CLSI) in 2022 [12].

### Phenotypic tests for carbapenemases

The modified carbapenem inactivation method (mCIM) and EDTA-modified carbapenem inactivation method (eCIM) were performed for phenotypic confirmation on the production of carbapenemases and further characterizing the types of carbapenemases. The tests were carried out according to the performance standards of antimicrobial susceptibility testing by CLSI in 2022 [12]. As all the isolates were already known to harbour *bla*_*NDM*_, both mCIM and eCIM were carried out in parallel.

### Tests for AZT and CAZ-AVI synergy

#### Broth microdilution method

BMD for synergy testing was performed in a flat-bottomed microtiter plate using Muller Hinton Broth (HiMedia Laboratories, India) as per the method described by Rawson et al. [13]. AZT’s minimum inhibitory concentrations (MIC) against the isolates were determined in the presence and absence of CAZ-AVI. The AZT concentrations varied from 0.5μg/ml to 256μg/ml in the wells of the microtiter plate. For synergy testing, the AVI concentration of 4 μg/ml and the CAZ concentration of 16 μg/ml were fixed at each well. After inoculating the bacterial isolates in the well, the microtiter plates were incubated overnight at 37◦ C. The AZT MIC was calculated as the lowest concentration of the antibiotic that inhibits visible growth of the tested isolate observed manually with the unaided eye. When growth occurred in all dilutions of the AZT, the MIC was recorded as greater than the highest concentration, i.e., >256 μg/ml. The MIC was recorded as less than or equal to the lowest concentration of the AZT when no growth occurred in any of the concentrations tested, i.e., ≤ 0.5 μg/ml. The following formula calculated the fractional inhibitory concentration (FIC) of AZT.

$${\text{FIC}}_{\text{AZT}}=\text{MIC}\;\text{of}\;\text{AZT}\;\text{in}\;\text{presence}\;\text{of}\;\text{CAZ-AVI}/\text{MIC}\;\text{of}\;\text{AZT}\;\text{alone}$$

Synergy was defined as a four-fold decrease in AZT MIC in the presence of AVI. Additionally, synergy was considered clinically relevant if the AZT MIC value in the presence of AVI reaches the CLSI AZT MIC breakpoint for the susceptible category in Enterobacterales, i.e., ≤ 4μg/ml.

#### E-strip disc diffusion method

**Fig 1:**
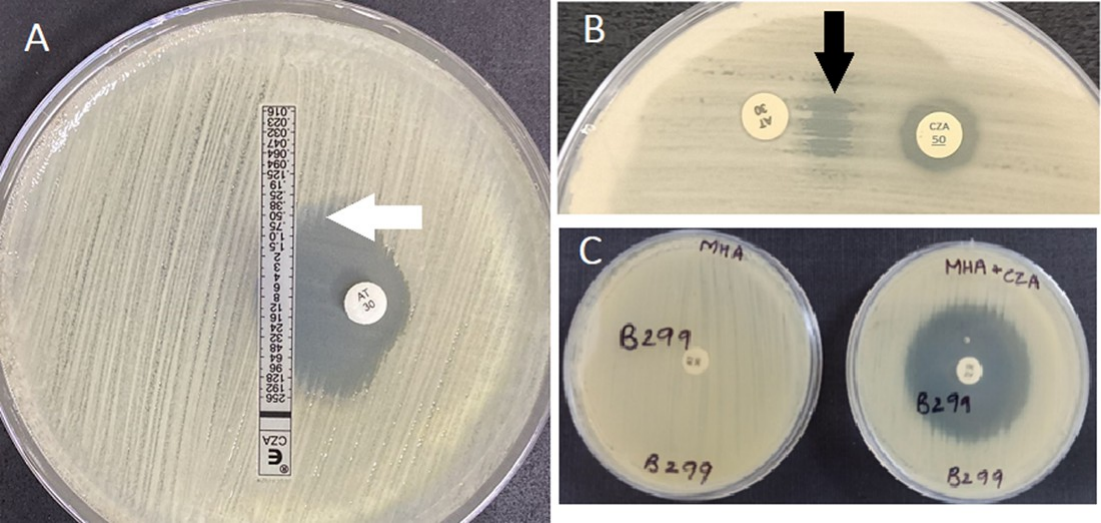
(A) Inverse D-shaped zone of inhibition (white arrow) depicting AZT and CAZ-AVI synergy in E-strip disc diffusion method. (B) Characteristic keyhole-shaped zone of inhibition (black arrow) depicting AZT and CAZ-AVI synergy in double disc diffusion method. (C) Supplemented disc diffusion method showing AZT zone of inhibition in CAZ-AVI supplemented MHA but no zone of inhibition for AZT in un-supplemented MHA.

A CAZ-AVI E-strip and AZT disc diffusion for synergy testing, described in the recent ICMR guidelines, was also performed [10]. A bacterial suspension matching 0.5 McFarland turbidity (measured with BD Phoenix Spec^™^ nephelometer, Becton Dickinson, USA) was inoculated onto Muller Hinton agar (MHA) plates by streaking with swab thrice over the entire surface and rotating the plate approximately 60 degrees each time to ensure even distribution of inoculums. A CAZ-AVI E-strip (bioMérieux, France) with a 4μg/ml fixed AVI concentration was placed on the agar surface. Then, an AZT disc (30 μg) was placed at a 1.5 cm distance from the CAZ-AVI E-test strip (centre to centre) near the susceptibility MIC breakpoint for Enterobacterales, i.e., ≤8/4μg/ml) as per CLSI criterion. Plates were incubated overnight at 37°C. Synergy by this method was interpreted by a qualitative approach of forming an inverse-D-shaped zone of inhibition (Fig 1A). Additionally, a quantitative approach was adopted to define synergy, as described by Rawson et al. [13]. AZT zone radiuses towards the CAZ-AVI E-strip and opposite to the CAZ-AVI strip were measured and converted to an estimated zone of inhibition diameters for AZT+AVI and AZT alone, respectively. Zone diameters were then compared as per CLSI zone diameter breakpoints and interpreted for synergy, based on restoration of AZT zone diameter (in the presence of AVI) crossing the CLSI susceptibility breakpoint, i.e., ≥ 21 mm [12].

#### Double disc diffusion method

MHA plate was inoculated with 0.5 McFarland turbid bacterial suspension. An AZT (30 μg) disc (HiMedia Laboratories, India) and a CAZ-AVI (30 μg /20 μg) disc (Becton Dickinson, USA) were placed on the seeded agar surface at a distance of 15 mm. The plate was then incubated overnight at 37°C.

Synergy was present if there was enhancement of the zone of inhibition of the AZT disc towards the CAZ-AVI disc and if there was a characteristic ’keyhole’ shaped zone of inhibition between two discs (Fig 1B).

#### Disc replacement method

MHA plate was inoculated with 0.5 McFarland turbid bacterial suspension. A CAZ-AVI (30 μg /20 μg) disc (Becton Dickinson, USA) was placed on the seeded agar surface, and the plate was incubated at 37°C for one hour. After that, the CAZ-AVI disc was quickly removed and replaced with an AZT (30 μg) disc at the same position on the agar surface. The plate was then further incubated overnight at 37°C [14].

Synergy was considered present if the zone diameter of the replacement AZT disc was ≥ 21 mm [12].

#### The proposed supplemented agar disc diffusion method

This method explored the ease of performing a simple disc diffusion test by ensuring uniform distribution of AVI in the agar. In Petri plates, the MHA medium was supplemented with CAZ-AVI (Pfizer, New York) (corresponding to an AVI concentration of 4μg/ml). The plates were inoculated (in a similar way followed in the standard disc diffusion test) from the same bacterial suspensions matching 0.5 McFarland turbidity used for the E-strip disc diffusion method. An AZT disc (30 μg) was placed on the supplemented MHA medium, and the medium was incubated overnight at 37°C. The AZT zone of inhibition diameter from the MHA plate supplemented with CAZ-AVI was then compared with the AZT zone of inhibition diameter from the standard disc diffusion plate with un-supplemented MHA, performed in parallel (Fig 1C). Interpretation of synergy was based on the restoration of AZT zone diameter (in the presence of AVI) crossing the CLSI susceptibility breakpoint, i.e., ≥ 21 mm [12].

### Interpretation and recording of results

Two independent primary observers read and interpreted the tests’ results individually. Any disagreement in interpreting the results between two observers was sorted with the interpretation by a third independent expert microbiologist with proficiency in reading and interpreting the results of the susceptibility testing (S1 Table).

### Statistical analysis

Agreement between the two methods or two primary observers was assessed with Cohen’s kappa (considered very good κ >0.75, good κ 0.40–0.75, and poor κ <0.40) [15]. The sensitivity and specificity of the synergy testing methods were determined using a 2 × 2 contingency table considering the BMD as the reference method.

## Results

A total of 37 carbapenem-resistant NDM-producing Enterobacterales (*Escherichia* coli 20/37, 54.05%; *K*. *pneumoniae* 13/37, 35.14%; *Enterobacter cloacae* 3/40, 8.11% & *Citrobacter braakii* 1/40, 2.7%) isolates were included in the study. The bacterial isolates were primarily obtained from pus/purulent fluids (14/37; 37.8%), followed by blood (11/37; 29.7%) and urine (7/37; 18.9%), respectively.

### Resistant profile of the isolates based on the standard disc diffusion method

All 37 isolates were resistant to imipenem, meropenem, ertapenem, and ceftazidime by standard disc diffusion. Two of the 37 isolates (5.4%) were tested sensitive to aztreonam; the other 35 (94.6%) isolates were aztreonam-resistant.

### Result of phenotypic test for carbapenemases

All isolates tested positive by both mCIM and eCIM. Hence, 100% of the isolates included in the study were phenotypically confirmed for production of MBL.

### Tests for AZT and CAZ-AVI synergy

#### Broth microdilution method

**Fig 2:**
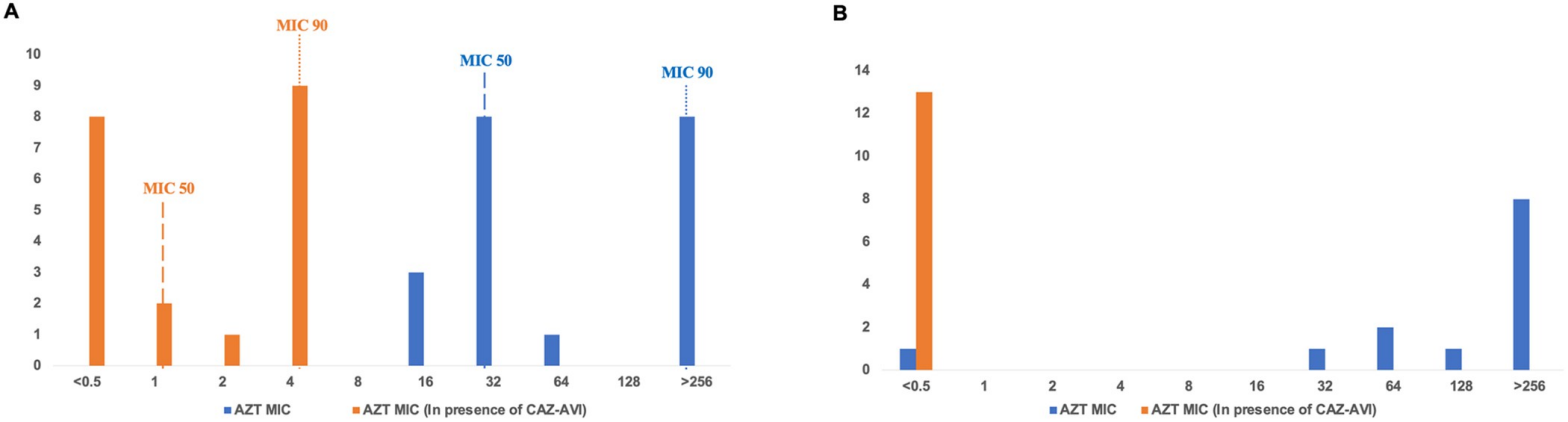
(A) Shifting of AZT MIC against *E*. *coli* in the presence of CAZ-AVI. (B) Shifting of AZT MIC against *K*. *pneumoniae* in the presence of CAZ-AVI.

The AZT MIC of the AZT-resistant isolates using the BMD method ranged from 16μg/ml to >256μg/ml (median 128μg/ml). In all 35 AZT-resistant isolates, ≥4-fold reduction in AZT MIC in the presence of AVI was observed, and these ranged from ≤0.5μg/ml to 4μg/ml (median ≤0.5μg/ml). Therefore, clinically relevant synergy was found in all AZT-resistant isolates (35, 100%). Adding AVI to AZT did not alter the MIC of two isolates, which were already AZT susceptible. Fig 2 depicts the shifting of AZT MIC towards the lower side in the presence of AVI in *E*. *coli* (A) and *K*. *pneumoniae* (B) isolates, respectively.

#### E-strip disc diffusion method

Synergy could be interpreted in 26/35 (74.3%) AZT-resistant isolates by either a qualitative or quantitative approach. In the remaining 9 (25.7%) isolates, the AZT zone of inhibition diameter (in the presence of AVI) remained below the zone diameter breakpoint for the susceptible category (as measured by the quantitative approach). Synergy was not demonstrable for the two AZT-susceptible isolates by either approach.

#### Double disc diffusion method

Out of the total 35 AZT-resistant isolates, synergy could be demonstrated in (21/35; 60%) AZT-resistant isolates by the double disc diffusion method.

#### Disc replacement method

Synergy was observed in 33/35 (94.28%) AZT-resistant isolates using the disc replacement method.

#### The proposed supplemented agar disc diffusion method

This method could reveal synergy in 34/35 (97.14%) AZT-resistant isolates. For the remaining isolate, the AZT zone of inhibition diameter (in the presence of AVI) was increased compared to that of AZT alone (20 mm in the presence of AVI compared to 15 mm for AZT alone). Still, it remained below the CLSI zone diameter breakpoint for the susceptible category (i.e., ≥ 21 mm) [12]. Table 1 depicts the changes in the AZT zone of inhibition in the presence of AVI alongside AZT MICs in the presence or absence of AVI in the reference BMD method for different isolates.

**Table 1:** Comparative analysis of the AZT MICs in the presence or absence of AVI (4 μg/ml) in the reference BMD method and changes in the aztreonam zone of inhibition in the supplemented agar disc diffusion method.

Isolate number	Organism	Broth microdilution method				Supplemented agar disc diffusion method		
		Aztreonam MIC μg/ml	Aztreonam MIC (in presence of ceftazidime-avibactam) μg/ml	Aztreonam FIC	Clinical synergy	Aztreonam zone diameter (un-supplemented MHA) mm	Aztreonam zone diameter (MHA supplemented with ceftazidime-avibactam) mm	Clinical synergy
B288	*E*.*coli*	>256	1	0.004	Present	No zone	25	Present
B307	*E*.*coli*	64	4	0.063	Present	10	21	Present
B71	*E*.*coli*	32	4	0.125	Present	15	23	Present
B105	*E*.*coli*	>256	≤0.5	0.002	Present	No zone	25	Present
B141	*E*.*coli*	>256	≤0.5	0.002	Present	No zone	35	Present
B157	*E*.*coli*	>256	≤0.5	0.002	Present	No zone	31	Present
B163	*E*.*coli*	>256	≤0.5	0.002	Present	No zone	35	Present
BC247	*K*.*pneumoniae*	>256	≤0.5	0.002	Present	No zone	30	Present
B167	*E*.*cloacae*	>256	≤0.5	0.002	Present	No zone	26	Present
U305	*E*.*coli*	32	4	0.125	Present	15	21	Present
B241	*K*.*pneumoniae*	64	≤0.5	0.008	Present	11	30	Present
BC288	*E*.*coli*	>256	≤0.5	0.002	Present	No zone	31	Present
B252	*E*.*coli*	32	≤0.5	0.016	Present	17	32	Present
BC422	*E*.*coli*	>256	≤0.5	0.002	Present	No zone	30	Present
BC453	*E*.*coli*	32	2	0.063	Present	14	23	Present
B299	*K*.*pneumoniae*	128	≤0.5	0.004	Present	No zone	32	Present
B339	*K*.*pneumoniae*	32	≤0.5	0.016	Present	14	34	Present
BC466	*E*.*coli*	32	≤0.5	0.016	Present	15	34	Present
B341	*E*. *cloacae*	64	≤0.5	0.008	Present	10	30	Present
BC584	*E*. *cloacae*	4	≤0.5	0.125	-	22	45	-
BC658	*E*.*coli*	32	4	0.125	Present	12	22	Present
B481	*E*.*coli*	32	4	0.125	Present	16	23	Present
BC707	*K*.*pneumoniae*	>256	≤0.5	0.002	Present	No zone	30	Present
U1213	*K*.*pneumoniae*	>256	≤0.5	0.002	Present	No zone	30	Present
U1269	*K*.*pneumoniae*	64	≤0.5	0.008	Present	10	40	Present
U1256	*K*.*pneumoniae*	>256	≤0.5	0.002	Present	No zone	27	Present
B576	*E*.*coli*	16	4	0.25	Present	17	23	Present
B567	*E*.*coli*	16	4	0.25	Present	17	22	Present
B584	*K*.*pneumoniae*	>256	≤0.5	0.002	Present	No zone	31	Present
U1257	*K*.*pneumoniae*	>256	≤0.5	0.002	Present	No zone	30	Present
B598	*C*.*braakii*	128	≤0.5	0.004	Present	8	45	Present
BC807	*E*.*coli*	16	4	0.25	Present	15	20	Absent
BC690	*E*.*coli*	32	4	0.125	Present	13	24	Present
U1186	*K*.*pneumoniae*	>256	≤0.5	0.002	Present	No zone	31	Present
BC735	*K*.*pneumoniae*	≤0.5	≤0.5	1	-	28	30	-
B578	*E*.*coli*	>256	1	0.004	Present	No zone	25	Present
U1317	*K*.*pneumoniae*	>256	≤0.5	0.002	Present	No zone	29	Present

Synergy was not demonstrable for the two AZT susceptible isolates, and the AZT zone of inhibition in the presence of AVI did not differ much and remained in the susceptible category.

### Comparative analysis of the methods for testing AZT and CAZ-AVI synergy

The sensitivity of the supplemented agar disc diffusion method, disc replacement method, E-test disc diffusion method and double disc diffusion method were 97.14%, 94.28%, 74.29%, and 60%, respectively [Table 2]. The specificity of all methods was 100%. The overall agreement between different methods and the reference BMD method was calculated based on Cohen’s kappa value. The highest agreement was observed for the supplemented agar disc diffusion method (κ = 0.78), followed by the disc replacement method (κ = 0.64), the E-strip disc diffusion method (κ = 0.23), double disc diffusion method (κ = 0.13), respectively.

**Table 2:** Performance of all methods for testing AZT and CAZ-AVI synergy with respect to the reference BMD method.

Test performance	Supplemented agar disc diffusion method	Disc replacement method	E-test disc diffusion method	Double disc diffusion method
Sensitivity	97.14%	94.28%	74.29%	60%
Specificity	100%	100%	100%	100%

The overall variations in interpreting the synergy between two primary observers for various tests have been depicted in the S1 Table. The highest agreement between two primary observers was found in the supplemented agar disc diffusion method (κ = 1), followed by the double disc diffusion method (κ = 0.94), E-strip disc diffusion method (κ = 0.93), disc replacement method (κ = 0.72), respectively.

**Fig 3:**
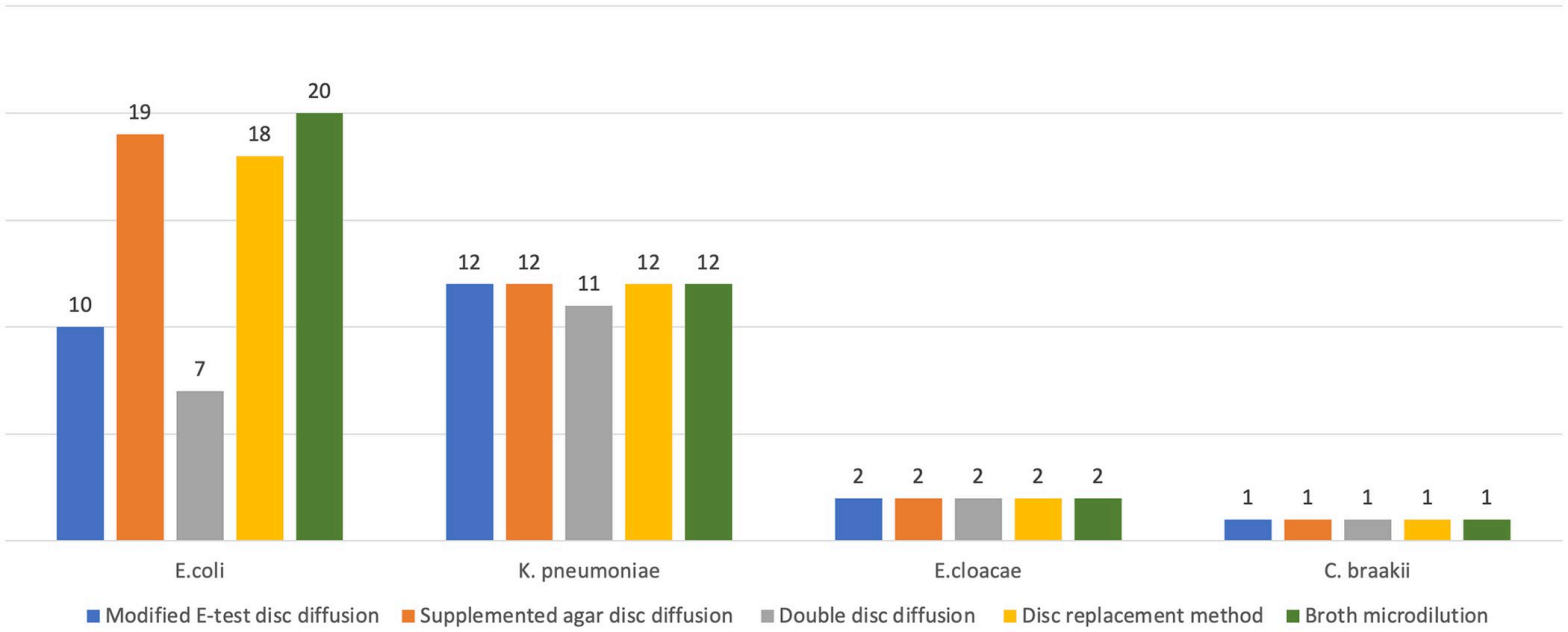
Comparison of different methods for testing AZT and CAZ-AVI synergy.

Fig 3 depicts the comparison among isolates belonging to different species in which various methods could detect AZT and CAZ-AVI synergy.

## Discussion

Infections caused by carbapenem-resistant organisms significantly challenge human health as the therapeutic options to treat these infections are limited. A reasonable treatment option for treating infections with Enterobacterales harbouring MBL like NDM with or without OXA-48 is the prolonged infusion of CAZ-AVI along with AZT [10]. In this triple combination, AZT helps in combating the MBL, whereas AVI (a novel beta-lactamase inhibitor) neutralizes the class A beta-lactamases (KPC, ESBL), class C beta-lactamases (AmpC) as well as some class D beta-lactamases (OXA-48-like), which are often co-produced in MBL producing organisms. CAZ, as such, has no role in the triple drug combination. Before using the above-mentioned drug combination in patients, it is imperative to test for in-vitro synergy of AZT in the presence of CAZ-AVI [10]. However, there is still no recommendation of a standard method for testing this synergy. Although some methods like broth disc elution, disc stacking, gradient strip stacking, and strip crossing have been proposed, searching for a simple testing method that may be readily implementable in a routine diagnostic microbiology laboratory continues. The present study evaluated a new, simple, and easily interpretable method and compared it with previously described methods.

In the present study, clinically relevant synergy with restoration of AZT MIC breakpoint in the presence of 4 μg/ml AVI could be demonstrated in all 35 AZT-resistant NDM-producing isolates. The mean FIC_AZT_ was 0.046 (range of 0.002 to 0.25). The FIC_AZT_ for all 35 AZT-resistant isolates were ≤0.5. Khan et al. also reported that FIC_AZT_ of ≤0.5 correlated well with AZT and CAZ-AVI synergy in the carbapenem-resistant Enterobacterales harbouring *bla*_*NDM*_ in their study [11].

Among all the synergy methods performed in our study, the proposed supplemented agar disc diffusion method had the highest agreement (κ = 0.78) with the reference BMD method, and it could detect the synergy in 34/35 (97.1%) total AZT-resistant isolates. On the other hand, the E-strip disc diffusion method, the disc replacement method, and the combined disc test methods could detect the synergy in 26/35 (74.3%), 33/35 (94.28%), and (21/35; 60%) AZT-resistant isolates, respectively. Rawson et al. reported that the E-strip disc diffusion method performed well by detecting the synergy in 93% (27/29) AZT-resistant isolates with 81% concordance with the BMD method in their study [13]. Conversely, Sahu et al. observed variable results of the E-strip disc diffusion method by which the synergy was demonstrated in 86% and 29% of *K*. *pneumoniae* and *E*. *coli* isolates, respectively [16]. In our study, the E-strip disc diffusion method results were variable across species, showing 100% synergy for *K*. *pneumoniae* but only 50% synergy for *E*. *coli* (Fig 3).

In our study, the disc replacement method could also detect the synergy in a comparatively high number of AZT-resistant (94.28%) isolates. Still, unlike the supplemented agar disc diffusion method, it required two-step processing, i.e., replacement of the CAZ-AVI disc with the aztreonam disc after an initial one hour of incubation. Sreenivasan et al. also reported 100% synergy for all Enterobacterales isolates using this method [14].

To the best of our knowledge, this is the first study to consider the interobserver variation between primary observers, interpreting the results of the synergy testing methods. The highest agreement between two primary observers was found to be best in the supplemented agar disc diffusion method (κ = 1), followed by the double disc diffusion method (κ = 0.94), E-strip disc diffusion method (κ = 0.93), disc replacement method (κ = 0.72), respectively. Therefore, the supplemented agar disc diffusion emerged as the best method for accuracy and lack of inter-observer variability. We assume that the consistency of the findings of the proposed method relies upon the design of the test. The basic principle for all methods for testing AZT and CAZ-AVI synergy is based on restoration of the AZT zone of inhibition diameter or AZT MIC in the presence of a fixed concentration of avibactam, i.e. 4μg/ml.

Interestingly, only the supplemented agar disc diffusion test and the reference BMD method ensure the fixed AVI concentration for testing the synergy, whereas the other tests used in the study involve diffusion of AVI from the disc or E-strip, resulting in unknown AVI concentration in the agar-based medium. Moreover, interpreting the results of the proposed method is easy and requires only measuring the AZT zone of inhibition diameter in the presence or absence of AVI. It also allows us to determine the true clinical synergy, i.e., the enhancement of the zone of diameter crossing the susceptibility breakpoint for AZT. However, interpreting the results of E-strip disc diffusion or combined disc diffusion methods may involve the qualitative approach by observing the formation of inverse D or characteristic keyhole, which may not ensure true clinical synergy. In addition to that, the test protocol of the supplemented agar disc diffusion method is technically less demanding, unlike the combined disc test or the E-strip disc diffusion, which requires meticulous positioning of the discs/ strips at a specified distance on the agar surface.

The present study also had a few limitations. Firstly, a relatively small number of Enterobacterales isolates were included in the study. These non-repetitive isolates were collected over a six-month period, which was specified for the present study. These isolates were carefully screened for carbapenem resistance and the presence of *bla*_*NDM*_ in their genome. Moreover, only the first isolate of a given species per patient collected within six months, irrespective of the specimen type, was considered to avoid bias in the study results. Secondly, the genomic analysis of the study involved the detection of only *bla*_*VIM*_, *bla*_*IMP*_, *bla*_*NDM*_, *bla*_*KPC*_, and *bla*_*OXA-48*_ determinants by the Xpert Carba R tests. Detection of any additional genes responsible for carbapenem resistance was beyond the scope of our study but could have helped us understand the genetic diversity of the isolates. Nevertheless, we performed phenotypic tests like mCIM and eCIM to confirm the production of MBL enzymes by the Enterobacterales isolates.

The simple supplemented agar disc diffusion method is user-friendly in routine microbiology laboratories to report in-vitro AZT and CAZ-AVI synergy against the difficult-to-treat MBL-producing carbapenem-resistant Enterobacterales. Clinical outcome-based studies with relatively large sample sizes will be beneficial in supporting our findings.

## Supporting information

S1 Table Inter-observer variation in determination of AZT and CAZ-AVI synergy with different methods. (DOCX)
