# Supplementary material for: Evaluation of a simple method for testing aztreonam and ceftazidime-avibactam synergy in New Delhi metallo-beta-lactamase producing Enterobacterales
Source: PLoS One. 2024 May 17;19(5):e0303753. doi: 10.1371/journal.pone.0303753 (PMC11101023; doi:10.1371/journal.pone.0303753)
Supplement: S1 Table — (DOCX) [file pone.0303753.s001.docx]

**S1 Table: Inter-observer variation in determination of AZT and CAZ-AVI synergy with different methods**

| **Isolate number** | **Synergy in modified E-test disc diffusion method** | | | **Synergy in supplemented agar disc diffusion method** | | | **Synergy in double disc diffusion method** | | | **Synergy in disc replacement method** | | |
| --- | --- | --- | --- | --- | --- | --- | --- | --- | --- | --- | --- | --- |
|  | **Observer 1** | **Observer 2** | **Final interpretation** | **Observer 1** | **Observer 2** | **Final interpretation** | **Observer 1** | **Observer 2** | **Final interpretation** | **Observer 1** | **Observer 2** | **Final interpretation** |
| B288 | Present | Present | Present | Present | Present | Present | Absent | Absent | Absent | Present | Present | Present |
| B307 | Absent | Absent | Absent | Present | Present | Present | Absent | Absent | Absent | Present | Present | Present |
| B71 | Absent | Absent | Absent | Present | Present | Present | Absent | Absent | Absent | Absent | Present | Present |
| B105 | Present | Present | Present | Present | Present | Present | Present | Present | Present | Present | Present | Present |
| B141 | Present | Present | Present | Present | Present | Present | Present | Present | Present | Present | Present | Present |
| B157 | Present | Present | Present | Present | Present | Present | Present | Present | Present | Present | Present | Present |
| B163 | Present | Present | Present | Present | Present | Present | Present | Present | Present | Present | Present | Present |
| BC247 | Present | Present | Present | Present | Present | Present | Present | Present | Present | Present | Present | Present |
| B167 | Present | Present | Present | Present | Present | Present | Present | Present | Present | Present | Present | Present |
| U305 | Absent | Absent | Absent | Present | Present | Present | Absent | Absent | Absent | Present | Present | Present |
| B241 | Present | Present | Present | Present | Present | Present | Present | Present | Present | Present | Present | Present |
| BC288 | Present | Present | Present | Present | Present | Present | Present | Present | Present | Present | Present | Present |
| B252 | Absent | Absent | Absent | Present | Present | Present | Absent | Absent | Absent | Present | Present | Present |
| BC422 | Present | Present | Present | Present | Present | Present | Absent | Absent | Absent | Present | Present | Present |
| BC453 | Absent | Absent | Absent | Present | Present | Present | Absent | Absent | Absent | Present | Present | Present |
| B299 | Present | Present | Present | Present | Present | Present | Absent | Absent | Absent | Present | Present | Present |
| B339 | Present | Present | Present | Present | Present | Present | Present | Present | Present | Present | Present | Present |
| BC466 | Present | Present | Present | Present | Present | Present | Absent | Absent | Absent | Present | Present | Present |
| B341 | Present | Present | Present | Present | Present | Present | Present | Present | Present | Present | Present | Present |
| BC584 | - | - | - | - | - | - | - | - | - | - | - | - |
| BC658 | Present | Absent | Absent | Present | Present | Present | Absent | Absent | Absent | Absent | Present | Absent |
| B481 | Absent | Absent | Absent | Present | Present | Present | Absent | Absent | Absent | Present | Present | Present |
| BC707 | Present | Present | Present | Present | Present | Present | Present | Present | Present | Present | Present | Present |
| U1213 | Present | Present | Present | Present | Present | Present | Present | Present | Present | Present | Present | Present |
| U1269 | Present | Present | Present | Present | Present | Present | Present | Present | Present | Present | Present | Present |
| U1256 | Present | Present | Present | Present | Present | Present | Present | Present | Present | Present | Present | Present |
| B576 | Present | Present | Present | Present | Present | Present | Absent | Absent | Absent | Present | Present | Present |
| B567 | Absent | Absent | Absent | Present | Present | Present | Present | Absent | Present | Present | Present | Present |
| B584 | Present | Present | Present | Present | Present | Present | Present | Present | Present | Present | Present | Present |
| U1257 | Present | Present | Present | Present | Present | Present | Present | Present | Present | Present | Present | Present |
| B598 | Present | Present | Present | Present | Present | Present | Present | Present | Present | Present | Present | Present |
| BC807 | Absent | Absent | Absent | Absent | Absent | Absent | Absent | Absent | Absent | Absent | Absent | Absent |
| BC690 | Present | Present | Present | Present | Present | Present | Absent | Absent | Absent | Present | Present | Present |
| U1186 | Present | Present | Present | Present | Present | Present | Present | Present | Present | Present | Present | Present |
| BC735 | - | - | - | - | - | - | - | - | - | - | - | - |
| B578 | Present | Present | Present | Present | Present | Present | Present | Present | Present | Present | Present | Present |
| U1317 | Present | Present | Present | Present | Present | Present | Present | Present | Present | Present | Present | Present |
